# Hydrogen-Rich Water Enhances Ibuprofen-Induced Antinociception and Modulates NOX Signaling and the NLRP3 Inflammasome in Chronic Inflammatory Pain

**DOI:** 10.3390/ijms27188093

**Published:** 2026-09-11

**Authors:** Giovanna Schiavoni, Weitao Wang, Sylmara Esther Negrini-Ferrari, Olga Pol

**Affiliations:** 1 Grup de Neurofarmacologia Molecular, Institut de Recerca Sant Pau (IR SANT PAU), Sant Quintí 77-79, 08041 Barcelona, Spain; 2 Grup de Neurofarmacologia Molecular, Institut de Neurociències, Universitat Autònoma de Barcelona, 08193 Barcelona, Spain

**Keywords:** molecular hydrogen, ibuprofen, NSAIDs, chronic inflammatory pain, oxidative stress, NLRP3 inflammasome, NADPH oxidase

## Abstract

Chronic inflammatory pain is sustained by persistent oxidative stress and inflammatory signaling that promote peripheral and central sensitization. Although nonsteroidal anti-inflammatory drugs (NSAIDs), including ibuprofen, remain first-line therapies, their long-term use is limited by dose-dependent adverse effects. Therefore, strategies capable of enhancing NSAID efficacy while maintaining an acceptable safety profile are of considerable therapeutic interest. In this study, we investigated whether hydrogen-rich water (HRW) enhances the antinociceptive effects of ibuprofen in male C57BL/6J mice with complete Freund’s adjuvant (CFA)-induced chronic inflammatory pain. Dose–response studies demonstrated that both HRW and ibuprofen significantly attenuated mechanical allodynia and thermal hyperalgesia. Notably, co-administration of a low dose of HRW with ibuprofen produced significantly greater antiallodynic and antihyperalgesic effects than either treatment alone. Molecular analyses further showed that the combined treatment reduced the paw expression of the oxidative stress marker 4-hydroxynonenal (4-HNE) and the inflammasome component NLRP3, as well as spinal NADPH oxidases (NOX), NOX1 and NOX4 expression, while maintaining the CFA-induced upregulation of heme oxygenase-1 (HO-1) and superoxide dismutase-1 (SOD-1) protein levels. In contrast, spinal NOX2 expression remained unchanged. These findings indicate that the enhanced antinociceptive effect of HRW plus ibuprofen is associated with coordinated modulation of oxidative stress and inflammatory pathways at peripheral and/or spinal levels. Collectively, our results support HRW as a promising adjuvant strategy for improving NSAID-mediated analgesia and provide a strong preclinical rationale for further mechanistic and translational investigation of this combination as a multimodal therapeutic approach for chronic inflammatory pain.

## 1. Introduction

Chronic pain is a complex and debilitating condition characterized by persistent pain that is often difficult to manage. Nonsteroidal anti-inflammatory drugs (NSAIDs), such as ibuprofen, are widely used in the management of chronic pain due to their well-established anti-inflammatory and analgesic properties. However, long-term NSAID therapy is associated with significant adverse effects, including gastrointestinal, renal, and cardiovascular toxicity [1,2]. Therefore, the development of alternative or complementary therapeutic strategies that provide effective analgesia with fewer side effects remains a critical clinical need.

Chronic inflammatory pain arises from complex interactions between the immune system, the nervous system, and intracellular molecular signaling pathways [3]. One of the key mechanisms involved is the activation of the transcription factor nuclear factor kappa B (NF-κB), which contributes to the priming and transcriptional upregulation of NLRP3 inflammasome components [4,5]. NLRP3 is a crucial component of a cytosolic multiprotein complex responsible for the maturation and release of interleukin-1β (IL-1β) [6,7]. This pro-inflammatory cytokine interacts with its receptors on sensory neurons, increasing ion channel permeability and lowering the action potential threshold, thereby inducing neuronal hyperexcitability. These changes drive both peripheral and central sensitization, contributing to the development of allodynia and hyperalgesia characteristic of chronic inflammatory pain [8].

In addition to inflammation, oxidative stress plays a pivotal role in the pathogenesis of chronic pain. Inflamed tissues generate elevated levels of reactive oxygen species (ROS), which promote lipid peroxidation and the formation of toxic aldehydes such as 4-hydroxynonenal (4-HNE) [9,10]. This highly reactive molecule forms adducts with cellular proteins, disrupts membrane integrity, and further enhances neuronal sensitization and nociceptive signaling [11]. To counteract oxidative damage, endogenous antioxidant defense pathways are activated, among which the nuclear factor erythroid 2–related factor 2 (Nrf2) pathway is particularly important. Nrf2 induces the expression of cytoprotective enzymes such as heme oxygenase-1 (HO-1), which exhibits antioxidant and anti-inflammatory properties relevant to chronic pain regulation [12], and superoxide dismutase-1 (SOD-1), which catalyzes the conversion of superoxide radicals into less reactive species, thereby limiting ROS-induced tissue damage [13]. Moreover, Nrf2 activation contributes to the suppression of pro-inflammatory signaling pathways, including NF-κB, which in turn may limit NLRP3 inflammasome assembly and cytokine production [12]. Together, these molecular actions may contribute to reducing neuronal hyperexcitability and central sensitization, key mechanisms in the initiation and maintenance of chronic pain states.

NADPH oxidases (NOX) are major enzymatic sources of ROS and are increasingly recognized as key mediators of chronic pain by promoting oxidative stress, neuroinflammation, and peripheral and central sensitization [14]. Among the NOX family, three isoforms (NOX1, NOX2, and NOX4) have been implicated in chronic pain mechanisms. Current evidence indicates that NOX1 is predominantly involved in inflammatory pain, whereas NOX2 and NOX4 have been more extensively implicated in neuropathic pain [15]. Genetic deletion of Nox1 or pharmacological inhibition with the selective inhibitor ML171 markedly attenuates nociceptive behaviors in several inflammatory pain models, including formalin- and carrageenan-induced pain [16,17]. In addition, ROS generated by NOX2-expressing macrophages have been shown to activate TRPA1 and subsequently stimulate NOX1 in Schwann cells, thereby amplifying peripheral sensitization [18]. Although NOX4 has been primarily linked to neuropathic pain, emerging evidence suggests that changes in NOX4 expression may also be associated with inflammatory pain by promoting spinal microglial activation through the NOX4/JAK2/STAT3/NLRP3 signaling pathway [19,20].

Molecular hydrogen (H_2_) has gained increasing attention as a novel therapeutic strategy for chronic pain due to its ability to reduce oxidative stress and inflammation, cross the blood–brain barrier, and exhibit a favorable safety profile with minimal reported side effects [21,22]. H_2_ exerts antinociceptive effects primarily through the modulation of oxidative and inflammatory pathways, as evidenced by reductions in lipid peroxidation products such as 4-HNE and decreased expression of inflammatory markers, including NLRP3, in several preclinical models of chronic pain [23,24,25]. Based on these findings, we hypothesized that HRW, through its anti-inflammatory and antioxidant properties [26,27], may provide protective benefits when combined with ibuprofen. Therefore, we postulated that the combination of HRW and ibuprofen would enhance analgesic efficacy compared with either treatment alone.

In this study, we investigated whether co-administration of HRW enhances the antinociceptive effects of ibuprofen in a preclinical model of chronic inflammatory pain induced by subplantar injection of complete Freund’s adjuvant (CFA). Specifically, we evaluated the dose–response effects of HRW and ibuprofen administered separately, assessed the efficacy of their combined treatment on mechanical allodynia and thermal hyperalgesia, and analyzed changes in oxidative stress markers (4-HNE, HO-1, and SOD-1) and the inflammatory marker NLRP3 in paw tissues. Furthermore, we evaluated the protein levels of NOX1, NOX2, and NOX4 in the spinal cord (SC) to determine whether the combined treatment modulates the expression of these NOX isoforms involved in chronic pain.

## 2. Results

### 2.1. CFA-Induced Mechanical Allodynia and Thermal Hyperalgesia

**Figure 1:**
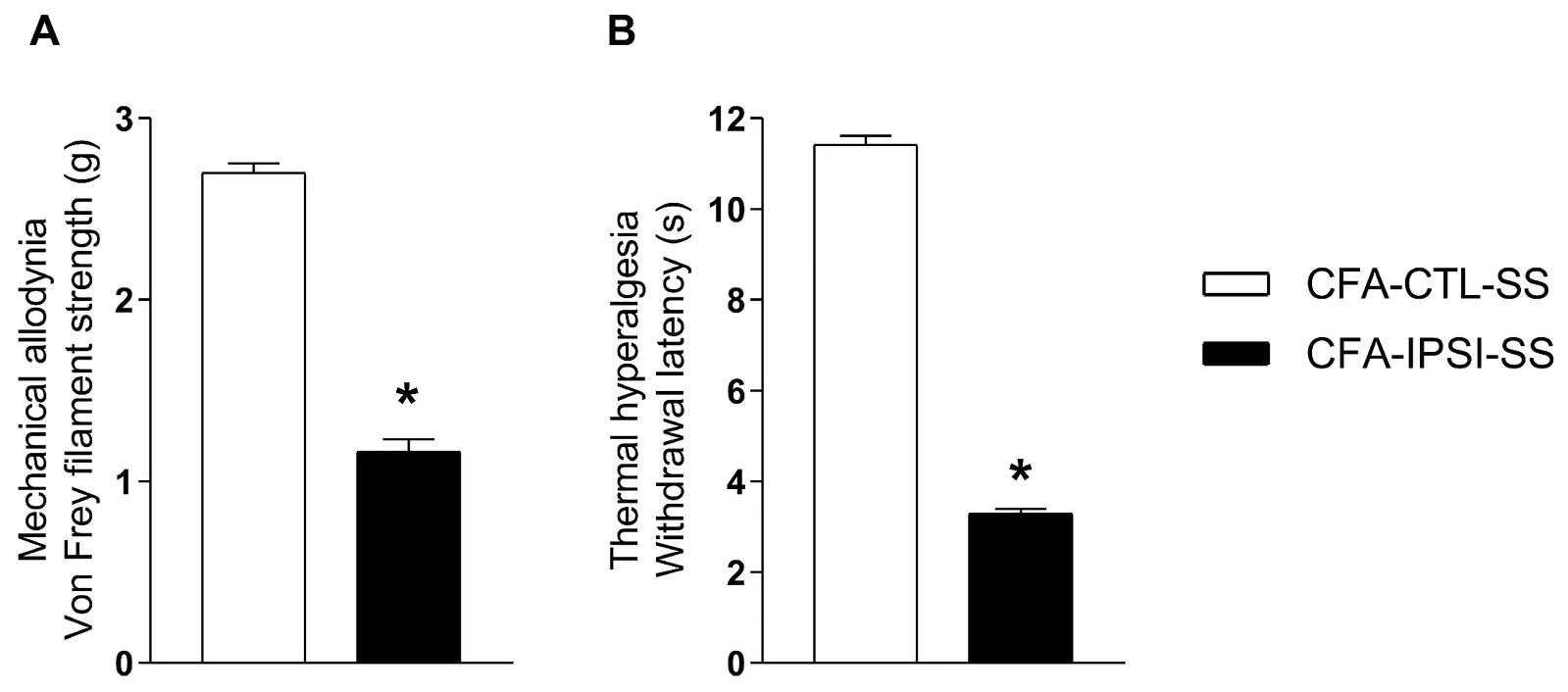
Assessment of nociceptive sensitivity 14 days after CFA injection. (**A**) Mechanical allodynia was evaluated using the von Frey test and is expressed as the paw withdrawal threshold (g). (**B**) Thermal hyperalgesia was assessed using the plantar test and is expressed as paw withdrawal latency (s). Measurements were obtained from the contralateral (CTL) and ipsilateral (IPSI) paws of CFA-injected mice. CFA administration significantly reduced both the mechanical withdrawal threshold and thermal withdrawal latency in the ipsilateral paw, indicating the development of mechanical allodynia and thermal hyperalgesia, respectively. For each test, * indicates *p* < 0.001 vs. contralateral paw (paired Student’s *t*-test). Data are presented as mean ± SEM (*n* = 6 mice).

To validate the establishment of the inflammatory pain model, nociceptive responses were evaluated 14 days after subplantar injection of CFA. Consistent with previous findings, CFA injection produced a marked unilateral increase in nociceptive sensitivity, evidenced by the development of both mechanical allodynia and thermal hyperalgesia (Figure 1).

As shown in Figure 1A, CFA significantly reduced the mechanical withdrawal threshold in the ipsilateral paw compared with the contralateral paw, indicating the presence of mechanical allodynia (*p* < 0.001, paired Student’s *t*-test). Similarly, CFA-injected mice exhibited a significant decrease in thermal withdrawal latency in the ipsilateral paw relative to the contralateral paw (Figure 1B), indicating thermal hyperalgesia (*p* < 0.001, paired Student’s *t*-test).

These findings confirm the successful induction of persistent inflammatory pain and support the use of this model for evaluating the antinociceptive effects of HRW and ibuprofen, administered alone or in combination.

### 2.2. Dose–Response Effects of HRW and Ibuprofen on CFA-Induced Allodynia and Hyperalgesia

**Figure 2:**
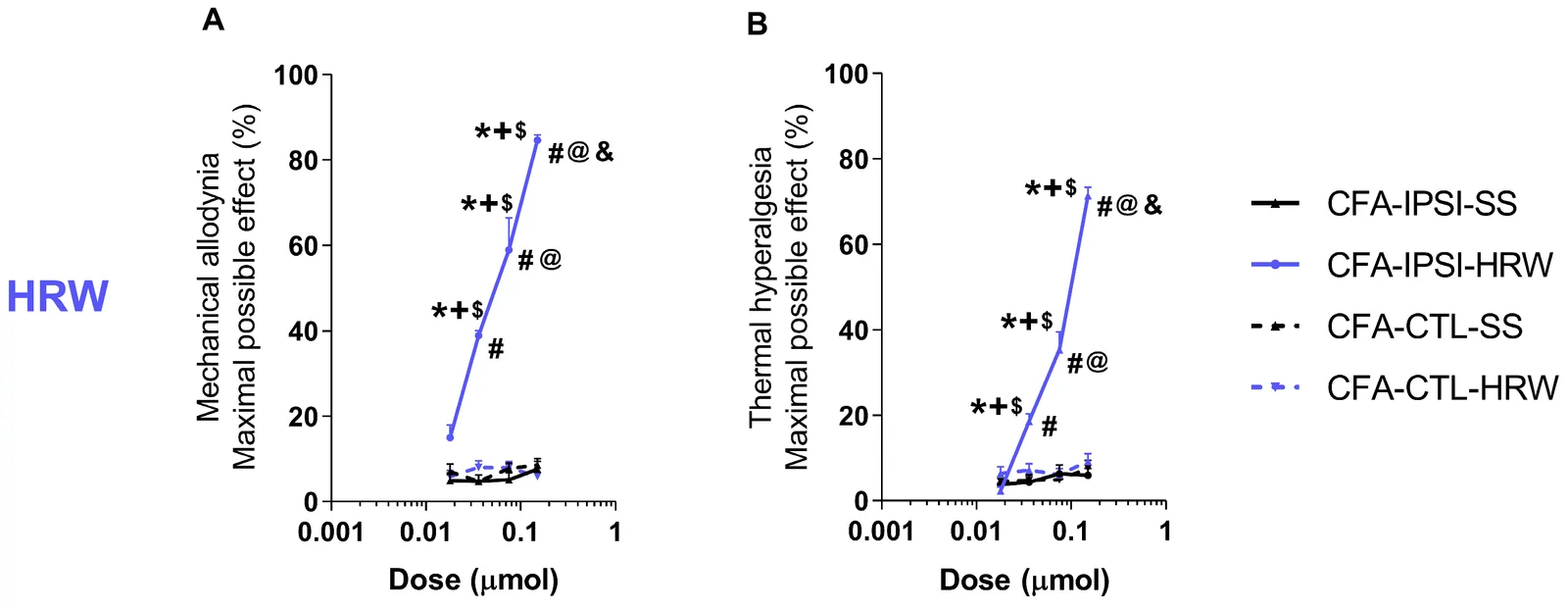
Dose-dependent antiallodynic and antihyperalgesic effects of HRW in CFA-induced inflammatory pain. Intraperitoneal administration of HRW (0.018, 0.036, 0.075, and 0.150 μmol) was evaluated in mice with CFA-induced peripheral inflammation. Antinociceptive responses are expressed as the percentage of the maximal possible effect (% MPE). Mechanical allodynia is shown in panel (**A**), and thermal hyperalgesia in panel (**B**). The *x*-axis represents the HRW dose on a logarithmic scale. Responses were assessed in the ipsilateral (IPSI) and contralateral (CTL) paws of CFA-injected animals. Symbols indicate significant differences: * vs. CFA-IPSI-SS; + vs. CFA-CTL-SS; $ vs. CFA-CTL-HRW; # vs. CFA-IPSI-HRW (0.018 μmol); @ vs. CFA-IPSI-HRW (0.036 μmol); and & vs. CFA-IPSI-HRW (0.075 μmol). Statistical analysis was performed using two-way repeated-measures ANOVA, with dose as the between-subject factor and paw (IPSI vs. CTL) as the within-subject factor, followed by Sidak’s multiple-comparisons test (*p* < 0.05). Data are presented as mean ± SEM (*n* = 6 mice per dose).

Intraperitoneal administration of HRW (0.018–0.150 μmol) produced dose-related antinociceptive effects in mice with CFA-induced inflammatory pain, significantly reducing both mechanical allodynia and thermal hyperalgesia (Figure 2A,B).

Two-way repeated-measures ANOVA revealed significant effects of HRW dose and paw (ipsilateral vs. contralateral), as well as a significant dose × paw interaction, for both mechanical allodynia (dose, *p* < 0.0021; paw, *p* < 0.0001; interaction, *p* < 0.0001) and thermal hyperalgesia (dose, paw, and interaction, all *p* < 0.0001).

Sidak’s multiple-comparisons test showed that HRW significantly increased the percentage of the maximal possible effect (% MPE) in the ipsilateral paw compared with saline-treated CFA mice. Specifically, doses of 0.036, 0.075, and 0.150 μmol produced significantly greater antiallodynic and antihyperalgesic effects than saline treatment in the ipsilateral paw, whereas no significant dose-related differences were observed in the contralateral paw.

In the mechanical allodynia test (Figure 2A), the antiallodynic effect increased progressively with dose. HRW at 0.150 μmol produced a significantly greater effect than all lower doses (0.018, 0.036, and 0.075 μmol). Moreover, 0.075 μmol produced a greater effect than 0.018 and 0.036 μmol, whereas 0.036 μmol was also more effective than 0.018 μmol.

A similar dose-dependent pattern was observed for thermal hyperalgesia (Figure 2B). HRW at 0.150 μmol produced a significantly greater antihyperalgesic effect than all lower doses. In addition, 0.075 μmol was more effective than 0.018 and 0.036 μmol, whereas 0.036 μmol produced a greater effect than 0.018 μmol.

The maximal antinociceptive effect of HRW was observed at 0.150 μmol, reaching approximately 84% and 70% of the MPE in the mechanical allodynia and thermal hyperalgesia tests, respectively.

**Figure 3:**
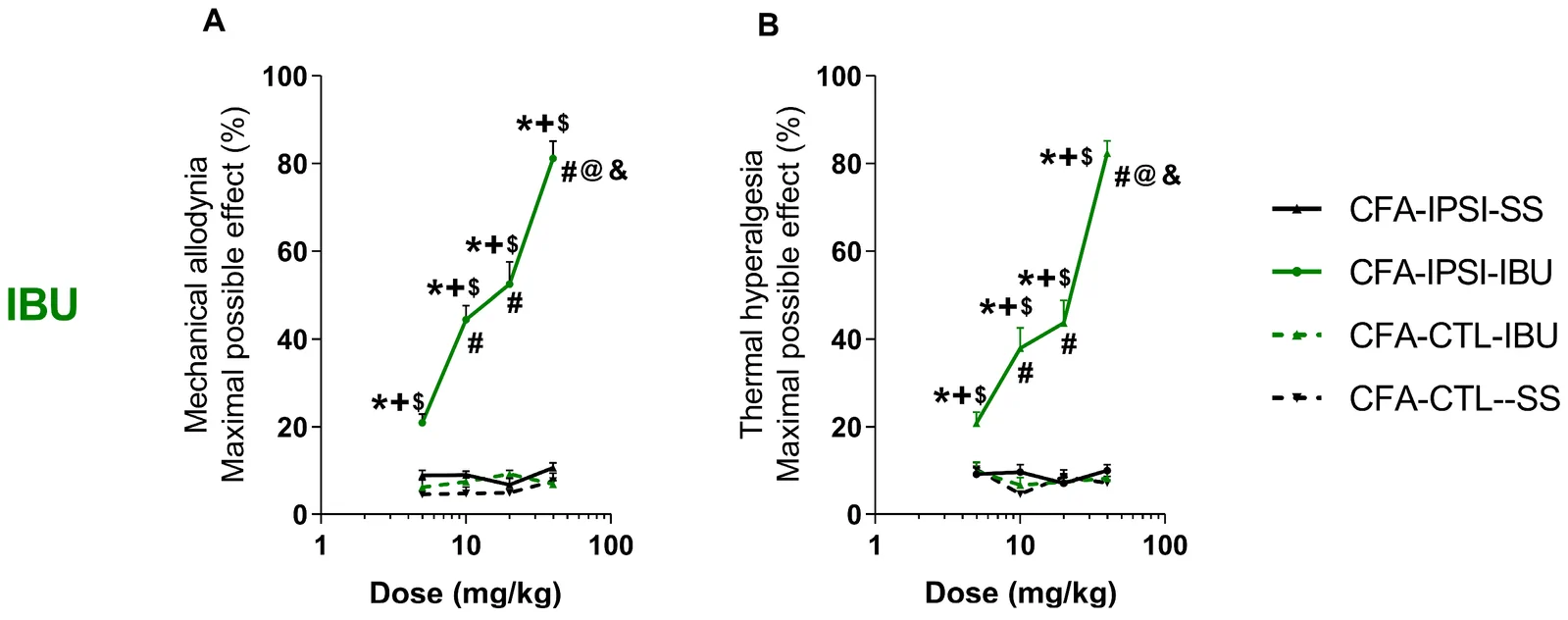
Dose-dependent antiallodynic and antihyperalgesic effects of ibuprofen in CFA-induced inflammatory pain. Oral administration of ibuprofen (IBU; 5, 10, 20, and 40 mg/kg) was evaluated in mice with CFA-induced peripheral inflammation. Antinociceptive responses are expressed as the percentage of the maximal possible effect (% MPE). Mechanical allodynia and thermal hyperalgesia are shown in panels (**A**,**B**), respectively. Ibuprofen doses are plotted on a logarithmic scale. Responses were assessed in the ipsilateral (IPSI) and contralateral (CTL) paws of CFA-injected animals. Symbols indicate significant differences: * vs. CFA-IPSI-SS; + vs. CFA-CTL-SS; $ vs. CFA-CTL-IBU; # vs. CFA-IPSI-IBU (5 mg/kg); @ vs. CFA-IPSI-IBU (10 mg/kg); and & vs. CFA-IPSI-IBU (20 mg/kg). Statistical analysis was performed using two-way repeated-measures ANOVA, with dose as the between-subject factor and paw (IPSI vs. CTL) as the within-subject factor, followed by Sidak’s multiple-comparisons test (*p* < 0.05). Data are presented as mean ± SEM (*n* = 6 mice per dose).

Oral administration of ibuprofen (5–40 mg/kg) produced dose-dependent antinociceptive effects in mice with CFA-induced inflammatory pain, reducing both mechanical allodynia and thermal hyperalgesia (Figure 3A,B).

Two-way repeated-measures ANOVA revealed significant effects of ibuprofen dose and paw (ipsilateral vs. contralateral) for both mechanical allodynia and thermal hyperalgesia (all *p* < 0.0001). Significant dose × paw interactions were also observed for mechanical allodynia (*p* < 0.0411) and thermal hyperalgesia (*p* < 0.0471).

Sidak’s multiple-comparisons test showed that all ibuprofen doses significantly increased % MPE in the ipsilateral paw compared with saline-treated CFA mice, whereas no significant dose-dependent effects were observed in the contralateral paw.

In the mechanical allodynia test (Figure 3A), the antiallodynic effect increased progressively with dose. Ibuprofen at 40 mg/kg produced a significantly greater effect than all lower doses (5, 10, and 20 mg/kg). In addition, 10 and 20 mg/kg produced significantly greater antiallodynic effects than 5 mg/kg, whereas no significant difference was detected between 10 and 20 mg/kg.

A similar dose-dependent pattern was observed for thermal hyperalgesia (Figure 3B). Ibuprofen at 40 mg/kg produced a significantly greater antihyperalgesic effect than all lower doses, whereas 10 and 20 mg/kg were significantly more effective than 5 mg/kg. No significant difference was detected between 10 and 20 mg/kg.

The maximal antinociceptive effect of ibuprofen was observed at 40 mg/kg, reaching approximately 81% and 82% of the MPE in the mechanical allodynia and thermal hyperalgesia tests, respectively.

### 2.3. Effects of Combined HRW and Ibuprofen Therapy on CFA-Induced Mechanical Allodynia and Thermal Hyperalgesia

**Figure 4:**
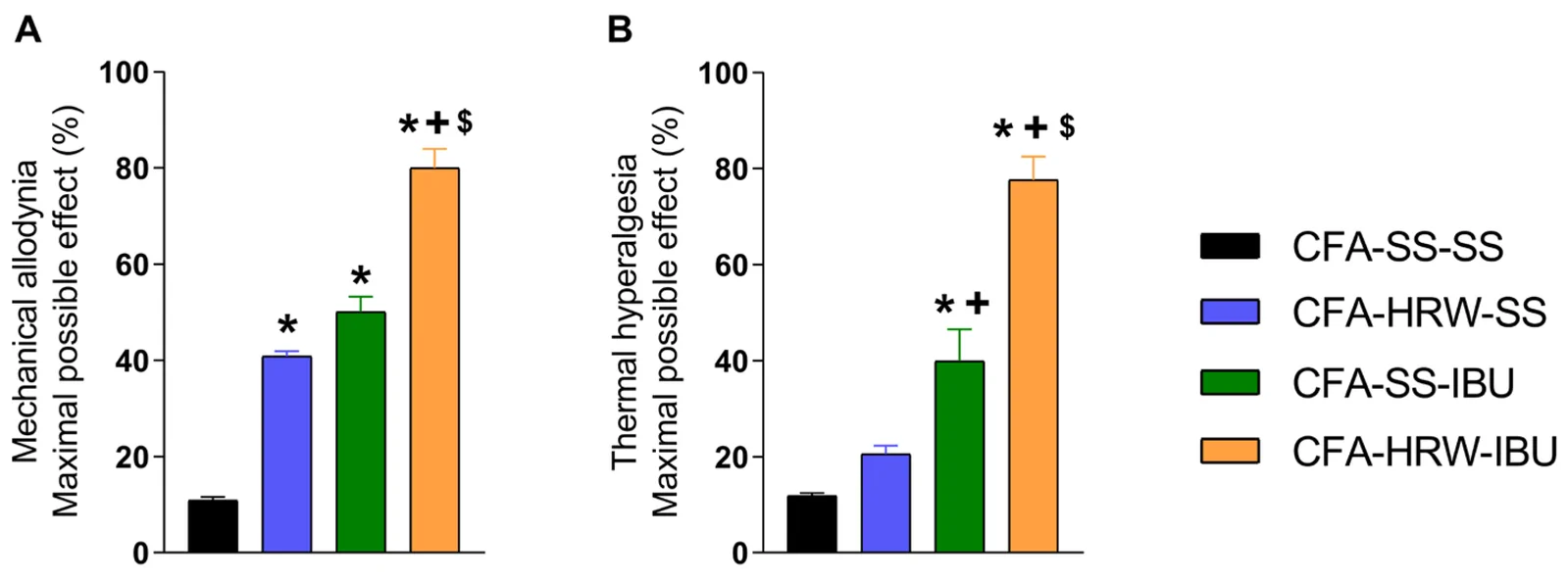
Combined antinociceptive effects of HRW and ibuprofen on CFA-induced inflammatory pain. Effects of a low dose of HRW (0.036 μmol, i.p.), ibuprofen (IBU; 20 mg/kg, p.o.), and their combined administration on CFA-induced mechanical allodynia (**A**) and thermal hyperalgesia (**B**) in the ipsilateral paw of mice. Antinociceptive responses are expressed as the percentage of the maximal possible effect (% MPE). Symbols indicate significant differences: * vs. CFA-SS-SS; + vs. CFA-HRW-SS; and $ vs. CFA-SS-IBU (*p* < 0.05; one-way ANOVA followed by Sidak’s post hoc test). Data are presented as mean ± SEM (*n* = 6 mice per group).

To determine whether HRW could enhance the antinociceptive effects of ibuprofen, the selected low active dose of HRW (0.036 μmol, i.p.) and an intermediate submaximal dose of ibuprofen (20 mg/kg, p.o.) were administered alone or in combination to mice with CFA-induced inflammatory pain (Figure 4).

As shown in Figure 4A, both HRW and ibuprofen administered alone significantly reduced CFA-induced mechanical allodynia compared with saline-treated animals. Notably, co-administration of HRW and ibuprofen produced a significantly greater antiallodynic effect than either treatment alone (*p* < 0.0001, one-way ANOVA), increasing the percentage of MPE to approximately 80%. No significant differences were observed between HRW and ibuprofen when administered individually.

A similar pattern was observed for thermal hyperalgesia (Figure 4B). Ibuprofen alone significantly reduced thermal hyperalgesia compared with saline-treated animals, whereas HRW alone produced only a modest effect that did not differ significantly from the saline-treated group. However, combined administration of HRW and ibuprofen produced a markedly greater antihyperalgesic response than either treatment alone, reaching approximately 78% of the MPE (*p* < 0.0001, one-way ANOVA). Ibuprofen alone produced a significantly greater antihyperalgesic effect than HRW alone.

Overall, these findings demonstrate that co-administration of HRW and ibuprofen produces greater antinociceptive effects in CFA-induced inflammatory pain than either treatment alone, resulting in enhanced antiallodynic and antihyperalgesic responses.

### 2.4. Impact of Combined HRW and Ibuprofen Therapy on Oxidative Stress and Inflammation in the Paw of CFA-Injected Mice

**Figure 5:**
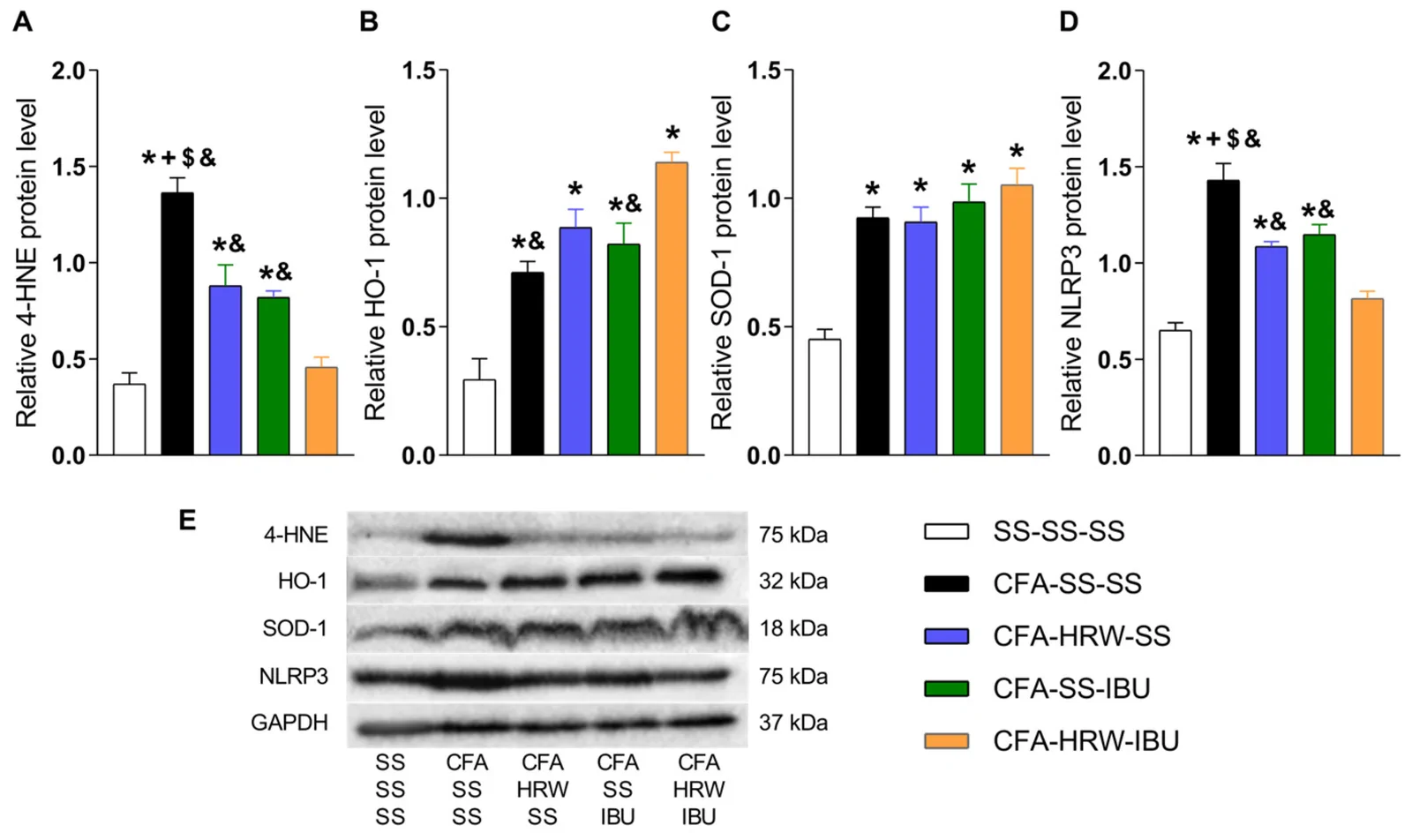
Effects of HRW and ibuprofen, administered alone or in combination, on oxidative stress- and inflammation-related markers in CFA-induced inflammatory pain. Protein expression of oxidative stress and inflammatory markers was evaluated in the ipsilateral paw tissues of CFA-injected mice treated with HRW (0.036 μmol, i.p.), ibuprofen (IBU; 20 mg/kg, p.o.), or their combined administration. Relative protein expression of 4-HNE (**A**), HO-1 (**B**), SOD-1 (**C**), and NLRP3 (**D**) was determined by Western blot and normalized to GAPDH. Representative immunoblots are shown in (**E**). The SS-SS-SS group was used as the non-inflamed control. Symbols indicate significant differences: * vs. SS-SS-SS; + vs. CFA-HRW-SS; $ vs. CFA-SS-IBU and & vs. CFA-HRW-IBU (*p* < 0.05; one-way ANOVA followed by Sidak’s multiple comparisons test). Data are presented as mean ± SEM (*n* = 6 samples per group).

The effects of HRW, ibuprofen, and their combined administration on oxidative stress and inflammatory responses associated with CFA-induced peripheral inflammation were evaluated in paw tissues. Protein expression of 4-HNE, HO-1, SOD-1, and NLRP3 was analyzed by Western blot (Figure 5).

As shown in Figure 5A, CFA administration significantly increased 4-HNE expression compared with the control group (SS-SS-SS) (*p* < 0.0001, one-way ANOVA). Treatment with either HRW or ibuprofen alone partially reduced 4-HNE levels, whereas their combined administration produced a greater reduction in 4-HNE expression than either treatment alone, resulting in protein levels comparable to those observed in the control group.

CFA-induced inflammation also significantly increased HO-1 and SOD-1 protein expression compared with the control group (*p* < 0.0001, one-way ANOVA; Figure 5B,C). The elevated expression of both antioxidant enzymes was maintained following treatment with HRW, ibuprofen, or their combination. In the case of HO-1, expression levels following combined HRW and ibuprofen treatment were also significantly higher than those observed after saline treatment or ibuprofen treatment alone.

NLRP3 protein expression was markedly increased in CFA-treated mice compared with the control group (*p* < 0.0001, one-way ANOVA; Figure 5D). Administration of HRW or ibuprofen alone significantly reduced CFA-induced NLRP3 expression, although levels remained significantly elevated relative to controls. Importantly, combined HRW and ibuprofen treatment produced a significantly greater reduction in NLRP3 expression than either treatment alone, resulting in NLRP3 levels similar to control values.

Overall, these findings indicate that combined HRW and ibuprofen treatment produced greater reductions in 4-HNE and NLRP3 expression, while maintaining the CFA-induced upregulation of HO-1 and SOD-1 protein expression.

### 2.5. Effects of HRW and Ibuprofen, Administered Alone or in Combination, on NOX Isoform Expression in the SC of CFA-Injected Mice

**Figure 6:**
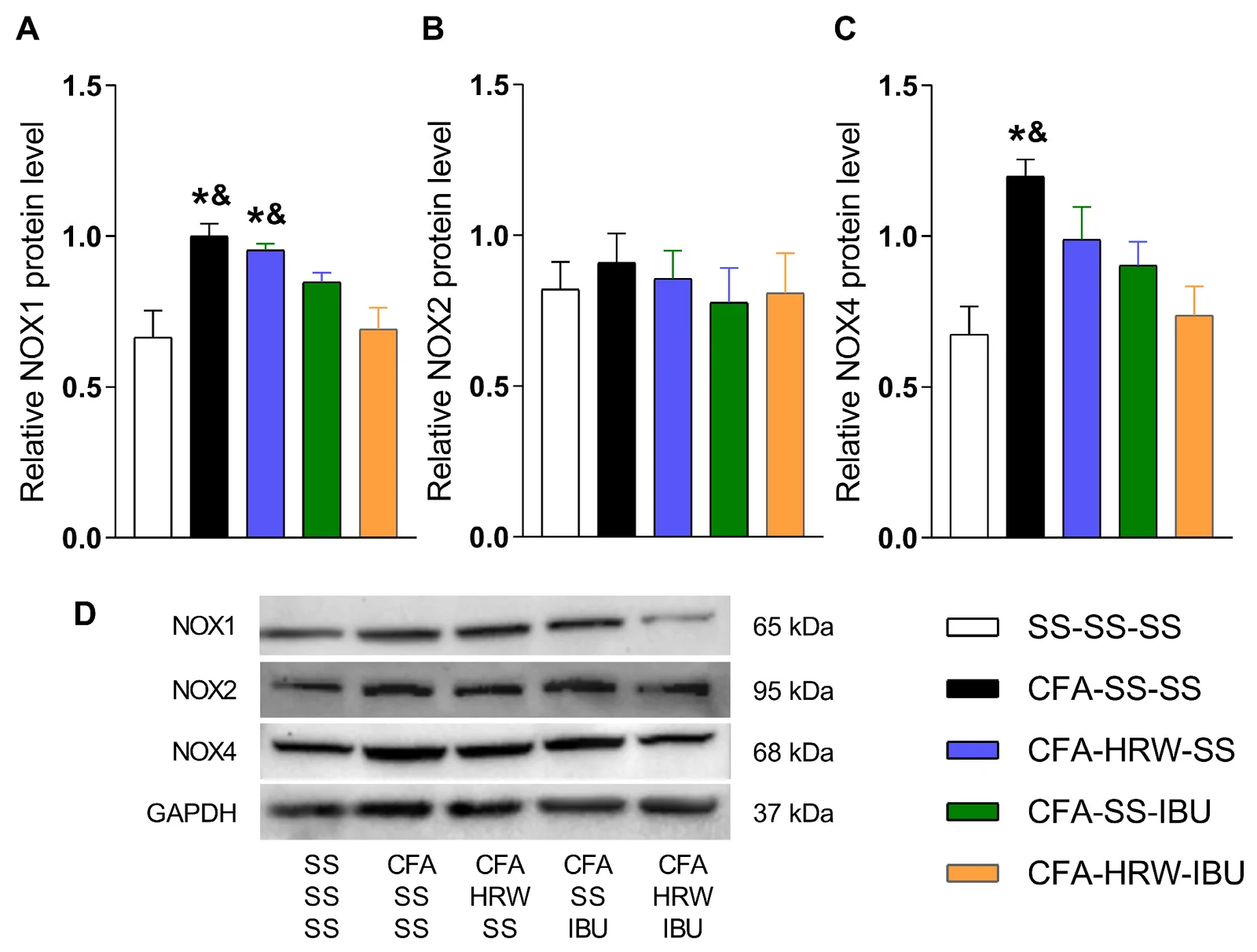
Effects of HRW and ibuprofen, administered alone or in combination, on NOX isoform expression in the SC of CFA-injected mice. Protein expression of NOX1, NOX2, and NOX4 was evaluated in the SC of CFA-injected mice treated with HRW (0.036 μmol, i.p.), ibuprofen (IBU; 20 mg/kg, p.o.), or their combined administration. Relative protein expression of NOX1 (**A**), NOX2 (**B**), and NOX4 (**C**) was determined by Western blot and normalized to GAPDH. Representative immunoblots are shown in (**D**). The SS-SS-SS group was used as the non-inflamed control. Symbols indicate significant differences: * vs. SS-SS-SS and & vs. CFA-HRW-IBU (*p* < 0.05; one-way ANOVA followed by Sidak’s multiple comparisons test). Data are presented as mean ± SEM (*n* = 6 samples per group).

To characterize changes in NOX isoform expression associated with the antinociceptive effects of HRW and ibuprofen, protein expression of NOX1, NOX2, and NOX4 was evaluated in the SC of CFA-injected mice (Figure 6).

CFA-induced inflammation significantly increased NOX1 (Figure 6A; *p* < 0.0010, one-way ANOVA) and NOX4 (Figure 6C; *p* < 0.0034, one-way ANOVA) protein expression compared with the non-inflamed control group (SS-SS-SS), whereas NOX2 expression remained unchanged among the experimental groups (Figure 6B). Combined administration of HRW and ibuprofen significantly attenuated CFA-induced upregulation of both NOX1 and NOX4, resulting in expression levels equivalent to those of the control group. In contrast, HRW or ibuprofen administered individually resulted in modest decreases in NOX1 and NOX4 expression that did not reach statistical significance.

## 3. Discussion

Our findings demonstrate that the co-administration of HRW and ibuprofen produces greater antinociceptive effects than either treatment alone. This enhanced effect was accompanied by reduced oxidative stress and inflammasome-related signaling while maintaining elevated protein expression of the antioxidant enzymes HO-1 and SOD-1, suggesting that HRW may represent a promising adjuvant strategy to improve the analgesic effects of ibuprofen in chronic inflammatory pain.

Consistent with previous studies in animals with inflammatory pain [28], ibuprofen produced dose-dependent antinociceptive effects, markedly attenuating both mechanical allodynia and thermal hyperalgesia induced by CFA. Likewise, HRW also exerted dose-dependent antiallodynic and antihyperalgesic effects, confirming and extending previous studies from our group demonstrating its analgesic activity in neuropathic pain models [24,25], and further demonstrating its dose-dependent effects in inflammatory pain. Importantly, both treatments produced no significant effects in the contralateral paws of CFA-injected mice, suggesting that their antinociceptive effects are preferentially expressed at the site of inflammation.

A major finding of this study is that the combination of HRW and ibuprofen produced greater antinociceptive effects than either treatment alone. Their co-administration resulted in greater inhibition of both mechanical allodynia and thermal hyperalgesia, supporting an enhanced antinociceptive effect of the combined treatment.

Similar potentiating effects of HRW have previously been reported in combination with HO-1 inducers, cannabinoids, gabapentinoids, and duloxetine in different neuropathic pain models [29,30,31]. However, to our knowledge, this is the first study to demonstrate enhanced antinociception following the combined administration of HRW and an NSAID in chronic inflammatory pain. Nevertheless, the greater antinociceptive effect observed with the combination should be interpreted as an enhanced response rather than as evidence of an additive or synergistic pharmacodynamic interaction, as formal interaction analyses and ibuprofen pharmacokinetic measurements were not performed.

Combination therapy is a well-established strategy in pain management, based on the premise that targeting complementary mechanisms may improve analgesic efficacy while potentially allowing lower doses of individual drugs and thereby limiting dose-related adverse effects [32]. This approach is particularly relevant in chronic pain conditions requiring prolonged pharmacological treatment. Conventional multimodal strategies to combat inflammatory pain include the combination of NSAIDs with opioids [33], whereas non-opioid approaches involving paracetamol or centrally acting agents such as duloxetine have also been investigated [34,35]. For example, naproxen combined with paracetamol showed additive analgesic effects in patients with rheumatoid arthritis [34], while the addition of duloxetine to NSAID therapy improved pain and physical function in patients with knee osteoarthritis inadequately controlled by NSAIDs [35]. Nevertheless, the benefits of the latter approach have not been consistently reproduced [36].

More recently, mechanism-based strategies involving drugs not traditionally used as analgesics have attracted increasing interest. Oral methotrexate added to usual analgesic therapy reduced pain and improved function in patients with osteoarthritis [37], whereas metformin has also emerged as a potential therapeutic strategy for osteoarthritis, with recent clinical and meta-analytic evidence suggesting beneficial effects on pain [38,39]. Nevertheless, these agents should be regarded as emerging therapeutic approaches rather than established NSAID combination therapies. Collectively, these findings illustrate the growing interest in multimodal strategies targeting distinct mechanisms involved in persistent pain, although their efficacy, tolerability, potential for drug interactions, and long-term safety remain important considerations [40].

In this context, HRW, characterized by its antioxidant and anti-inflammatory actions with minimal reported side effects [21,22], may offer a distinct pharmacological advantage by complementing the analgesic action of ibuprofen with modulation of mechanisms involved in oxidative stress and inflammation. In the present study, the greater antinociceptive actions of the combined treatment were accompanied by reduced oxidative and inflammatory markers while preserving the elevated expression of endogenous antioxidant defenses. Specifically, the combined administration of HRW and ibuprofen produced a greater attenuation of oxidative stress than either treatment alone. CFA-induced inflammation markedly increased paw 4-HNE expression, indicative of enhanced lipid peroxidation, whereas the combined treatment resulted in 4-HNE levels comparable to those observed in non-inflamed controls. Interestingly, despite reducing oxidative damage, the combination also maintained the CFA-induced upregulation of HO-1 and SOD-1 protein expression. Nevertheless, because enzymatic activity and overall antioxidant capacity were not directly measured in the present study, these findings should be interpreted as evidence of preserved endogenous antioxidant-related protein expression rather than increased antioxidant enzymatic activity or capacity.

In support of our findings, pharmacological activation of antioxidant pathways has been associated with reduced oxidative stress and nociception in experimental models of inflammatory pain. In CFA-induced inflammatory pain, dimethyl fumarate, an Nrf2 activator, reduced oxidative damage and inflammation while enhancing Nrf2/HO-1 signaling and antioxidant defenses [41]. Moreover, pharmacological induction of HO-1 has been shown to attenuate inflammatory pain through the activation of antioxidant pathways and modulation of NLRP3 inflammasome signaling [42]. Thus, the preservation of elevated HO-1 and SOD-1 expression, together with the reduction in 4-HNE levels induced by the combination of HRW and ibuprofen, may reflect a favorable redox environment in which pathological lipid peroxidation is attenuated without compromising endogenous antioxidant defenses. This effect may contribute to the greater antinociceptive effects observed with the combined treatment.

A particularly relevant finding of the present study is the differential modulation of NOX isoforms in the SC. CFA-induced inflammatory pain increased NOX1 and NOX4 expression, whereas NOX2 expression remained unchanged. Notably, the combined administration of HRW and ibuprofen significantly reduced the CFA-induced upregulation of both NOX1 and NOX4, restoring their expression to control levels.

The involvement of NOX1 in inflammatory pain is well established, as genetic deletion of *Nox1* attenuates mechanical and thermal hyperalgesia, while pharmacological inhibition of NOX1 reduces nociceptive sensitization by suppressing ROS-dependent signaling pathways [16,17]. In contrast, NOX4 has been predominantly associated with neuropathic pain, where it contributes to ROS production and persistent pain following peripheral nerve injury [15,19]. Our findings provide additional evidence of an association between spinal NOX4 expression and chronic inflammatory pain, as CFA-induced inflammation was associated with increased spinal NOX4 protein expression. Consistent with this observation, Yu et al. (2020) [20] reported increased NOX4 expression in the SC of CFA-treated mice, accompanied by activation of the JAK2/STAT3 pathway and the NLRP3 inflammasome. Given that NOX enzymes are major enzymatic sources of ROS, the concomitant downregulation of NOX1 and NOX4 observed following combined HRW and ibuprofen treatment may be associated with the attenuation of oxidative stress observed with the combined treatment. This effect may, in turn, limit ROS-dependent inflammatory signaling and thereby contribute to the enhanced antinociceptive effects of the combined treatment.

Activation of the NLRP3 inflammasome contributes to inflammatory pain by promoting the maturation and release of IL-1β and amplifying downstream inflammatory signaling [20,43,44]. Accordingly, inhibition of NLRP3 signaling has been associated with reduced inflammasome activation and pro-inflammatory cytokine production, together with attenuation of hypersensitivity in experimental models of neuropathic pain [45,46]. Consistent with these findings, the combined administration of HRW and ibuprofen produced the greatest reduction in NLRP3 expression.

ROS, including those generated by NOX enzymes, are important signaling molecules involved in NLRP3 inflammasome activation [47]. Thus, the concomitant downregulation of NOX1, NOX4, and NLRP3 observed in the present study is consistent with an association between reduced NOX-related oxidative signaling and reduced NLRP3 expression, which may contribute to the enhanced antinociceptive effects of the HRW–ibuprofen combination. However, the present findings do not establish a causal or functional contribution of NOX4 to these effects.

Previous studies from our group have shown that repeated administration of HRW alone attenuates chronic pain through complementary antioxidant and anti-inflammatory mechanisms, including activation of the Nrf2/HO-1 pathway, reduction of oxidative stress, and modulation of neuroinflammatory signaling under chronic pain conditions [23,24,25]. The present findings extend these observations by showing that acute co-administration of HRW and ibuprofen produces a greater reduction in NLRP3 expression than either treatment alone. Together with the concomitant downregulation of spinal NOX1 and NOX4, these results suggest a potential interplay between reduced NOX-derived oxidative signaling and attenuation of inflammasome-related pathways. Although the precise molecular mechanisms underlying this interaction remain to be fully elucidated, these complementary actions may contribute to the greater antinociceptive effect observed with the combined treatment.

Some limitations of this study should be acknowledged. First, the experiments were conducted in a single preclinical model of chronic inflammatory pain and included only male mice, which may limit the generalizability of the findings. Second, the molecular mechanisms proposed here were inferred from changes in protein expression rather than directly established using selective pharmacological inhibitors or genetically modified animals. Further investigation of the specific contributions of individual NOX isoforms and NLRP3 signaling would help establish their causal involvement in the enhanced effects of the HRW–ibuprofen combination. In addition, enzymatic activities of HO-1 and SOD-1 and overall antioxidant capacity were not directly measured; therefore, changes in their protein expression should not be interpreted as evidence of increased enzymatic activity or antioxidant capacity.

Another limitation is the absence of pharmacokinetic measurements of ibuprofen. Plasma and tissue concentrations of ibuprofen were not determined in the presence or absence of HRW. Therefore, we cannot exclude the possibility that HRW influences ibuprofen absorption, distribution, metabolism, or tissue exposure. Finally, the mechanistic studies were performed using a single HRW–ibuprofen dose combination. Evaluation of additional dose combinations and treatment regimens, together with formal pharmacological interaction analyses, would help define the optimal therapeutic regimen and clarify the nature of the interaction between the two treatments.

In conclusion, the present study demonstrates that HRW enhances the antinociceptive effects of ibuprofen in a mouse model of chronic inflammatory pain. Co-administration of HRW and ibuprofen produced greater antiallodynic and antihyperalgesic effects than either treatment alone and was associated with attenuation of oxidative and inflammatory alterations, including reduced 4-HNE and NLRP3 expression in the inflamed paw and downregulation of spinal NOX1 and NOX4, while maintaining elevated protein expression of HO-1 and SOD-1 in the paw.

These findings suggest that HRW may complement the pharmacological actions of ibuprofen by modulating oxidative and inflammatory pathways involved in persistent pain. Such complementary effects support the potential use of HRW as an adjuvant to NSAID therapy and raise the possibility that HRW may have dose-sparing potential when used in combination with ibuprofen.

Overall, our findings provide a preclinical rationale for further investigation of HRW as an adjunctive strategy for the treatment of chronic inflammatory pain. Further studies using oral HRW administration will be necessary to determine the translational potential of this combination.

## 4. Materials and Methods

### 4.1. Animals

Six- to eight-week-old male C57BL/6J mice obtained from Envigo Laboratories (Barcelona, Spain) were used in this study. Animals were maintained under a 12 h light/dark cycle at 22 °C and 66% relative humidity, with food and water available ad libitum. Mice were allowed to acclimatize for 7 days before the experiments. Experimental procedures were performed between 9:00 a.m. and 5:00 p.m. in accordance with the European Commission’s Directive (2010/63/EC) and the Spanish Law (RD 53/2013) regulation on animal experimentation. They were also authorized by the local Committee of Animal Use and Care of the Autonomous University of Barcelona (ethical code: 4581; date of approval: 31 March 2023).

Four male mice per cage were housed in polypropylene cages equipped with a cardboard shelter and cellulose bedding to provide environmental enrichment. Prior to testing, animals were acclimated to the experimental room for 1 h. For each experimental session, the order of testing was randomized daily, and each animal was evaluated at a different time on each test day. Two investigators were involved in the procedures for each animal: the primary investigator administered the treatment according to the randomization schedule and was the only individual aware of group allocation, while a second investigator, blinded to treatment, assessed mechanical allodynia and thermal hyperalgesia. Data analysis was also performed by an investigator blinded to group allocation.

All efforts were made to minimize animal suffering and to reduce the number of animals used, in accordance with the 3Rs principles (replacement, reduction, and refinement). Animals were randomly assigned to the experimental groups (*n* = 6 per group) using a randomization sequence generated with the RAND () function in Microsoft Excel. In total, 126 mice were used in this study.

All animals that completed the experimental protocol were included in the analysis. Animals showing signs of illness, improper CFA injection, or technical problems during behavioral procedures would have been excluded; however, no animals met any of these exclusion criteria in the present study.

Animals were monitored daily for general health and welfare, including changes in appearance, posture, mobility, grooming, body condition, and signs of pain or distress. Humane endpoints were predefined according to the approved animal experimentation protocol. Animals reaching any of these criteria would have been euthanized; nevertheless, no animals reached a humane endpoint during the study.

### 4.2. Induction of Inflammatory Pain

Inflammatory pain was induced by a subplantar injection of 30 μL of CFA (Sigma-Aldrich, St. Louis, MO, USA) into the right hind paw (ipsilateral). All injections were performed under brief isoflurane anesthesia (3% for induction and 2% for maintenance). The left hind paw (contralateral, CTL) was used as an internal control.

### 4.3. Assessment of Nociceptive Responses

Mechanical allodynia was evaluated using von Frey filaments (North Coast Medical, Inc., Morgan Hill, CA, USA). Animals were placed individually in Plexiglas cylinders (20 cm high × 9 cm diameter) positioned on an elevated wire mesh grid and allowed to acclimate for 30 min. Filaments were applied perpendicularly to the plantar surface of each hind paw until slight bending occurred. Testing began with the 0.4 g filament, and subsequent filament strength was increased or decreased according to the up–down method described by Chaplan et al. (1994) [48], depending on the presence or absence of a nociceptive response (paw withdrawal, shaking, or licking). A cutoff force of 3.0 g was established. Data analysis, including curve fitting, was performed using Microsoft Excel (Microsoft Iberia SRL, Barcelona, Spain).

Thermal hyperalgesia was evaluated using the plantar test apparatus (Ugo Basile, Varese, Italy). Mice were placed individually in Plexiglas cylinders (20 cm high × 9 cm diameter) positioned on a glass surface to measure paw withdrawal latency in response to a radiant heat stimulus. After a 30 min acclimatization period, the heat source was applied beneath each hind paw, and withdrawal latency was recorded with a cutoff time of 12 s to prevent tissue damage. Three measurements were obtained per paw at 5 min intervals, and the mean of these values was used as the paw withdrawal latency.

Both ipsilateral and contralateral hind paws were evaluated in both behavioral nociceptive tests.

### 4.4. Pharmacological Treatment

HRW was freshly prepared using an electrolysis generator (Hydrogen, Osmostar Soriano S.L., Alicante, Spain) at a stock concentration of 0.3 mM (300 µmol/L) in ultrapure Milli-Q water [23]. To minimize H_2_ dissipation, fresh HRW was generated individually for each animal and collected into a gas-tight glass syringe under zero-headspace conditions. Dissolved H_2_ concentration in the stock solution was verified immediately before administration at 25.0 ± 0.1 °C using a Clark-type microelectrode (H2-N, Unisense A/S, Aarhus, Denmark) housed in a 0.5 mL glass flow-through cell (1.2 mL/min), calibrated via a 2-point protocol (N_2_-purged vs. H_2_-saturated water).

Animals (*n* = 6 per group) received intraperitoneal injections of 0.018, 0.036, 0.075, or 0.150 µmol H_2_, delivered by transferring precise volumetric aliquots (60, 120, 250, and 500 µL, respectively) from the validated 0.3 mM stock solution using calibrated positive-displacement micropipettes. Stock concentration drift remained <3% throughout the procedure.

Ibuprofen, acquired from Sigma-Aldrich (St. Louis, MO, USA), was diluted in saline solution (NaCl 0.9%; SS) and orally administered at doses of 5, 10, 20 and 40 mg/kg (*n* = 6 animals per dose) in a final volume of 10 mL/kg.

Based on the dose–response results, a low active dose of HRW (0.036 μmol) and an intermediate, submaximal dose of ibuprofen (20 mg/kg) were selected for the combination experiments. These doses provided measurable antinociceptive effects while maintaining sufficient dynamic range to detect a potential enhancement of the response and avoiding the near-maximal effects observed at the highest doses.

All drugs were prepared immediately before use, and for each drug-treated group, the corresponding control group received the same volume of the respective vehicle (VEH). In accordance with previous studies [23,49], HRW and ibuprofen were administered 1 h before behavioral testing. HRW was administered intraperitoneally, based on our previous work demonstrating antinociceptive effects of systemically administered HRW in the same CFA-induced inflammatory pain model [23]. This route was maintained in the present study to ensure consistency with the previously validated experimental paradigm and to facilitate the rapid administration of freshly prepared HRW, thereby minimizing potential H_2_ loss prior to administration. Ibuprofen was administered orally consistent with its conventional route of administration.

The sample size was calculated using G*Power 3.1.7 software based on pilot data. Mechanical allodynia, assessed using the von Frey test, was considered the primary outcome for the sample-size calculation. Assuming a two-tailed test with an α error probability of 0.05 and a β error probability of 0.20 (corresponding to 80% statistical power), the analysis indicated that six animals per group were sufficient to detect statistically significant differences. Every effort was made to minimize animal suffering and reduce the number of animals used. Accordingly, tissues collected from animals subjected to behavioral testing were also used for Western blot analyses.

### 4.5. Experimental Design

**Figure 7:**
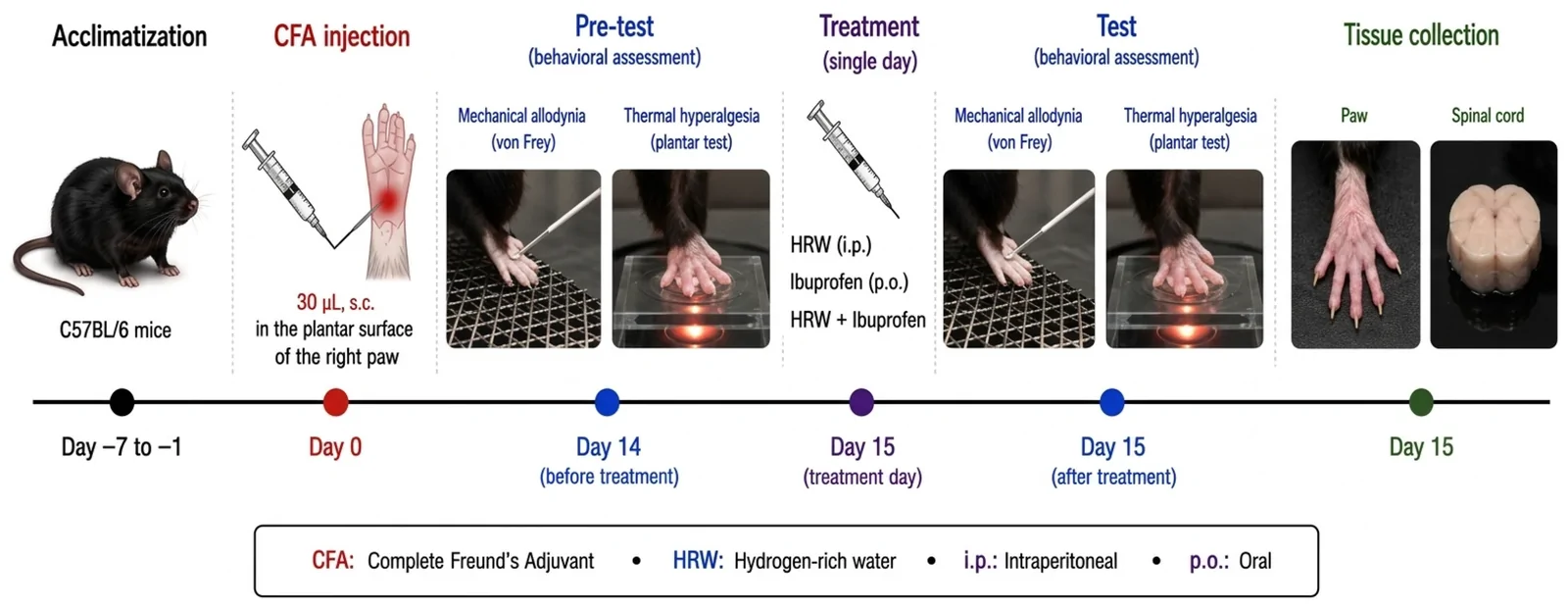
Experimental timeline of the CFA-induced inflammatory pain model and drug administration. The diagram illustrates the sequence, timing, and duration of the experimental procedures, including acclimatization, CFA injection, behavioral assessments, drug treatments, and tissue collection.

A mouse model of CFA-induced peripheral inflammation was used in this study. Mechanical allodynia and thermal hyperalgesia were evaluated at two time points: before CFA injection (baseline) and 14 days after CFA injection in both hind paws (*n* = 6 animals per group). As illustrated in Figure 7, the experimental timeline began with a 7-day acclimatization period, during which animals were allowed to adapt to the housing conditions. After acclimatization, baseline behavioral assessments were performed, consisting of the von Frey filament test to assess mechanical sensitivity and the plantar test to evaluate thermal nociception.

On day 0, CFA was injected into the right hind paw to induce peripheral inflammation. On day 14 post-CFA injection, behavioral tests were repeated to confirm the presence of mechanical allodynia and thermal hyperalgesia. On the following day (day 15), animals were treated with HRW, ibuprofen, or their combination at the doses specified in the corresponding treatment section (*n* = 6 animals per group).

Immediately after completion of the behavioral tests, animals were euthanized by cervical dislocation, and subplantar paw tissues and SC samples (L3–L6) were collected. Tissue samples were subsequently processed for Western blot analysis to evaluate changes in oxidative stress and inflammatory markers.

### 4.6. Protein Extraction and Western Blot Analysis

At 15 days after CFA injection, animals were euthanized by cervical dislocation, and subplantar tissues from the hind paws and SC (L3–L6) were rapidly collected, immediately frozen, and stored at −80 °C. Tissue homogenization was performed by finely dissecting the samples with sterile scissors, followed by the addition of ice-cold RIPA lysis buffer (Sigma-Aldrich, St. Louis, MO, USA) supplemented with protease (0.5%) and phosphatase (1%) inhibitor cocktails. Samples were then sonicated for 10 s and incubated at 4 °C for 1 h. Subsequently, homogenates were centrifuged at 700× *g* for 20 min at 4 °C, and the resulting supernatants were collected. Total protein concentration was determined using the Bradford assay.

For Western blot analysis, 60 μg of total protein was mixed with 4× Laemmli loading buffer and denatured at 95 °C for 5 min. Proteins were separated by SDS–PAGE using gels composed of a 4% stacking gel and a 12% resolving gel. After electrophoresis, proteins were transferred onto polyvinylidene difluoride (PVDF) membranes at 100 V for 100 min. Membranes were then blocked for 75 min at room temperature in phosphate-buffered saline (PBS) or Tris-buffered saline containing Tween-20 (TBST), supplemented with either 5% nonfat dry milk or 5% bovine serum albumin (BSA), to prevent nonspecific binding.

Subsequently, membranes were incubated overnight at 4 °C with the appropriate primary antibodies diluted in the corresponding blocking solution. The following primary antibodies were used: anti-4-HNE (1:150; AB46545; Abcam, Cambridge, UK), anti-HO-1 (1:200; ADI-SPA-895; Enzo Life Sciences, Farmingdale, NY, USA), anti-SOD-1 (1:150; NBP2-24915; Novus Biologicals, Littleton, CO, USA), anti-NLRP3 (1:200; AG-20B-0014-C100; AdipoGen Life Sciences, Epalinges, Switzerland), anti-NOX1 (1:150; 17772-1-AP), anti-NOX2 (1:150; 19013-1-AP), and anti-NOX4 (1:150; 14347-1-AP) (Proteintech, Rosemont, IL, USA), and anti-glyceraldehyde-3-phosphate dehydrogenase (GAPDH; 1:5000; AB516; Merck, Billerica, MA, USA) as a loading control.

Membranes were washed and then incubated for 1 h at room temperature with horseradish peroxidase (HRP)-conjugated anti-rabbit or anti-mouse secondary antibodies (GE Healthcare, Little Chalfont, UK). Protein bands were visualized using an enhanced chemiluminescence detection system (ECL kit; GE Healthcare) and quantified by densitometric analysis using ImageJ software (version 1.8.0; National Institutes of Health, Bethesda, MD, USA).

### 4.7. Statistical Analysis

All data are presented as mean ± standard error of the mean (SEM). Statistical analyses were performed using GraphPad Prism software (version 8 for Windows, La Jolla, CA, USA) and the Statistical Package for the Social Sciences (SPSS; version 28, IBM, Madrid, Spain). Assumptions underlying the statistical analyses were evaluated using Bartlett’s test for homogeneity of variances and the Shapiro–Wilk test to assess data normality.

Comparisons of CFA-induced changes in withdrawal thresholds to mechanical stimulation (von Frey filaments) and withdrawal latencies to thermal stimulation (plantar test) between ipsilateral and contralateral paws were performed using paired Student’s *t*-tests.

For the dose–response experiments, antiallodynic and antihyperalgesic effects were analyzed using two-way repeated-measures ANOVA, with treatment dose as the between-subject factor and paw (ipsilateral vs. contralateral) as the within-subject factor. When significant main effects or interactions were detected, Sidak’s multiple-comparisons test was applied. This approach accounted for the paired nature of ipsilateral and contralateral measurements obtained from the same animal.

For the combined-treatment experiments, antiallodynic and antihyperalgesic effects in the ipsilateral paw were analyzed using one-way ANOVA followed by Sidak’s multiple-comparisons test.

In the von Frey filament and plantar tests, antinociception was expressed as the percentage of the MPE, calculated from the pre-drug and post-drug responses according to the following formula:

% MPE = [(post-drug value − pre-drug value)/(cut-off value − pre-drug value)] × 100.

Cut-off values of 3 g and 12 s were used for mechanical allodynia and thermal hyperalgesia, respectively.

Changes in protein expression levels of 4-HNE, HO-1, SOD-1, NLRP3, NOX1, NOX2, and NOX4 were analyzed using one-way ANOVA followed by Sidak’s multiple-comparisons test.

A *p* value < 0.05 was considered statistically significant.

## 5. Conclusions

The present study demonstrates that HRW enhances the antinociceptive effects of ibuprofen in a mouse model of chronic inflammatory pain. Combined treatment produced greater antiallodynic and antihyperalgesic effects than either treatment alone and was associated with changes in oxidative stress- and inflammation-related protein expression, as evidenced by decreased NOX1, NOX4, and NLRP3 expression, together with maintenance of endogenous antioxidant defense protein expression. These findings support the potential of HRW as an adjuvant to conventional NSAID therapy and provide a preclinical rationale for further translational investigation of this combination in chronic inflammatory pain.

## Data Availability

The data presented in this study are available within the article. Additional data are available from the corresponding author upon reasonable request.
